# Staying at work with chronic nonspecific musculoskeletal pain: a qualitative study of workers' experiences

**DOI:** 10.1186/1471-2474-12-126

**Published:** 2011-06-03

**Authors:** Haitze J de Vries, Sandra Brouwer, Johan W Groothoff, Jan HB Geertzen, Michiel F Reneman

**Affiliations:** 1 Department of Rehabilitation Medicine, Center for Rehabilitation, University Medical Center Groningen, University of Groningen, The Netherlands; 2 Department of Health Sciences, Community and Occupational Medicine, University Medical Center Groningen, University of Groningen, The Netherlands

## Abstract

**Background:**

Many people with chronic nonspecific musculoskeletal pain (CMP) have decreased work ability. The majority, however, stays at work despite their pain. Knowledge about workers who stay at work despite chronic pain is limited, narrowing our views on work participation. The aim of this study was to explore why people with CMP stay at work despite pain (motivators) and how they manage to maintain working (success factors).

**Methods:**

A semi-structured interview was conducted among 21 subjects who stay at work despite CMP. Participants were included through purposeful sampling. Interviews were audio-recorded, transcribed verbatim, and imported into computer software Atlas.ti. Data was analyzed by means of thematic analysis. The interviews consisted of open questions such as: "Why are you working with pain?" or "How do you manage working while having pain?"

**Results:**

A total of 16 motivators and 52 success factors emerged in the interviews. Motivators were categorized into four themes: work as value, work as therapy, work as income generator, and work as responsibility. Success factors were categorized into five themes: personal characteristics, adjustment latitude, coping with pain, use of healthcare services, and pain beliefs.

**Conclusions:**

Personal characteristics, well-developed self-management skills, and motivation to work may be considered to be important success factors and prerequisites for staying at work, resulting in behaviors promoting staying at work such as: raising adjustment latitude, changing pain-coping strategies, organizing modifications and conditions at work, finding access to healthcare services, and asking for support. Motivators and success factors for staying at work may be used for interventions in rehabilitation and occupational medicine, to prevent absenteeism, or to promote a sustainable return to work. This qualitative study has evoked new hypotheses about staying at work; quantitative studies on staying at work are needed to obtain further evidence.

## Background

Chronic nonspecific musculoskeletal pain (CMP) is a prominent public-health problem in most welfare states. The influence of CMP on the degree of employee absenteeism and disability allowances is high [1-3]. However, the majority (60-70%) of workers with CMP stays at work despite pain and without sick leave [3-6]. This is not unique for CMP, for in cases of chronic disorders such as rheumatoid arthritis, diabetes mellitus, or COPD and asthma, most people also stay at work [7,8]. Understanding workers who stay at work despite pain is limited. Qualitative research on staying at work (SAW) has focused primarily on successful working strategies for women with fibromyalgia [9,10]. However, a qualitative study of SAW in working men and women with CMP is not yet available.

Up until now the subjects of absenteeism, work disability, and return to work (RTW) have dominated health and work research, in view of the large expenditure of funds by both society and employers, and, in addition, the personal problems of the employees who are on sick leave. Nevertheless, in spite of decades of extensive research focusing on absenteeism and RTW, no drastic changes in absenteeism and work participation levels have been identified. Disregarding in the literature the amount of people who stay at work might have limited our view on work participation. Knowledge of workers staying at work despite pain may be found useful for research and for the clinical practice of (vocational) rehabilitation, strategies for sustainable RTW, and occupational and insurance medicine. Effectiveness of vocational rehabilitation programs could be improved as soon as the success factors for SAW become clear. We may be able to learn from the successful workers' perspective, and identify factors that are essential for staying at work. Other authors agree that further exploration into this underreported and unknown group is needed [11,12].

Taken into account that knowledge on workers staying at work despite their pain is limited, a qualitative research approach was chosen as starting point for exploration into our research question [13]. This design is meant to offer a deeper understanding of perceived success factors for SAW despite CMP. It is relevant to know about the experiences of these workers, why they have decided to continue working with pain and how they have managed to be successful. What advice could their colleagues, who have not been able to stay at work, be given? Have they given up on other domains of participation? Which contributing factors could lead to being successful in working with chronic pain? Therefore, the aim of this study is to explore the motives of people with CMP in terms of why they stay at work despite pain, and the success factors of remaining working.

## Methods

### Study design

Individual semi-structured interviews were conducted. A qualitative study design was chosen, because it elucidates data from the experiences of the workers themselves, thus opening up the study to authentic themes, independent from prevailing constructs, instruments, or questionnaires. Thematic analysis was used to analyze the data [14].

### Subjects

Semi-structured interviews were conducted among 21 subjects with CMP (9 male, 12 female) who stayed at work despite CMP. These subjects were sampled from participants in the study "Working with Pain" which was conducted from May 2009 to January 2010. Participants in the "Working with Pain" study were recruited through announcements in newspapers and websites of national associations for Whiplash and Fibromyalgia patients. Inclusion criteria of the "Working with Pain" study were: CMP, duration longer than 6 months; age 20 to 60 years; having been employed 20 hours a week or more during 12 months prior to participation in the study; and participants' absence from work ascribed to CMP could not be more than 5% of potential total working hours in the year prior to participation (which is around the average rate of sickness absence in Europe) [15-17]. Exclusion criteria were: relevant co-morbidities with severe negative consequences for physical and/or mental functioning (for example severe mental illness), addiction to drugs, pregnancy, and insufficient knowledge of the Dutch language. To diagnose the type of pain and the existence of co-morbidities, all participants received a standard medical examination by a physiatrist. Sick leave was recorded by a standard questionnaire constructed by Rehabilitation Development Centers in the Netherlands [18].

To answer the review question and fully understand the topic, workers from various settings were interviewed. A purposeful sampling strategy was used to ensure that the sample consisted of a rich mixture of different perspectives according to gender, age, social background, and occupation [13]. The characteristics of the interview participants (n = 21) have been outlined in Table 1. Pain intensity was measured using the 11-point numeric rating scale (NRS), ranging from 0 (no pain) to 10 (worst possible pain), requiring participants to rate their current pain intensity and average pain intensity [19]. Validity and utility of the 11-point NRS is sufficient and it is responsive to changes in individuals [20-22]. The Pain Disability Index (PDI) was used to measure the degree of chronic pain interfering with daily life [23]. The PDI is a 7-item inventory, with each item score ranging from 0 (no interference) to 10 (total interference). The total PDI score ranges from 0 to 70. Reliability and validity of the PDI are supported by the literature [24].

**Table 1:** Demographic characteristics of 21 interview participants

Variable	mean (sd)	n	%
Age	49 (6.9)		
20-30 years		0	
31-40 years		4	19
41-50 years		4	19
51-60 years		13	62
Gender			
Male		9	43
Female		12	57
Education			
Primary		8	38
Secondary		6	29
Higher		7	33
Profession			
Teacher		4	19
Healthcare		6	29
Sales		2	10
Engineering		3	14
Gardening		3	14
Administration		2	10
Journalist		1	4
Working hours	31 (8.4)		
20 hours		4	19
21-30 hours		5	24
31-40 hours		12	57
Pain location			
(low) Back		9	43
Neck/shoulders		7	33
Fibromyalgia		5	24
Pain intensity NRS ^a ^now	4.5 (1.9)		
1-4		8	38
5-7		9	43
8-10		4	19
Pain intensity NRS average ^b^	5.3 (1.7)		
1-4		2	10
5-7		12	57
8-10		7	33
Pain duration			
1-2 years		3	14
3-5 years		0	0
>5 years		18	86
Pain Disability Index (PDI)	20.3 (8.2)		
0-10		2	10
11-20		7	33
21-30		10	47
>30		2	10
Occupational parameter PDI	3.7 (1.5)		
Work			
Paid employment		19	90
Self-employed		2	10
Work absence previous year ^c^			
0 days		17	81
1-10 days		4	19

^a ^Numeric Rating Scale

^b ^Average pain rating during the last 7 days

^c ^Work absence due to chronic musculoskeletal pain

The study was judged and approved by the Medical Ethical Committee of the University Medical Center of Groningen. Anonymity, confidentiality, and the right to withdraw from the study at all times were guaranteed. All participants signed an informed consent form.

### The interview

Large numbers of explanatory models and theories have been constructed to understand and explain sick leave and work attendance [25-32]. Each model seems to have shortcomings. For that reason, no explicit theoretical framework was used in the construction of the interview so as to enable participants to speak for themselves without theoretical constraints imposed by the interviewer and also to set no limitations on the interviewer's mindset. We have used open questions in our interview such as, "Why are you working with pain?" and "How do you manage working with your pain?" Topics of relevance were developed at an expert meeting attended by occupational, rehabilitation, and insurance physicians; a healthcare psychologist; an expert on labor; and a patient representative. Topics included were motivators for SAW, success factors for SAW, coping strategies promoting SAW, future expectations regarding work participation, what can be learned from workers who stay at work, as well as consequences of SAW (Additional file 1). These topics were tested in a trial by way of seven interviews, after which the interview guide was altered slightly, and some new topics were added (Additional file 1: Questions 4, 17, 18, 20, and 29). The interview guide guaranteed that no information was overlooked, whereas the semi-structured format also made allowances for spontaneous interaction. After completion of the pilot study, the sampling and interviewing of the participants started.

### Data analysis

Interviews lasted 45-90 minutes, and were audio-recorded and transcribed verbatim. The first three interviews were transcribed by the interviewer (HdV), while the rest were done by a secretary. The transcribed text was verified and corrected by the interviewer. Data was analyzed according to the theoretical approach of the method of thematic analysis [14]. Atlas.ti computer software was used for data analysis. To find answers to our research questions, the interview texts were analyzed, guided by the themes "why" and "how." The analyses were completed by the interviewer in close collaboration with the second author (SB). At first, the transcribed interviews were read and open-coded by the first and second author, independently. The research questions "*why*" and "*how*" guided the coding process. Agreement was reached on the naming and defining of the preliminary emerged codes. An experienced psychologist was consulted about the coding. After rereading the interviews, codes were renamed, combined, or split, and classified by themes. Peer debriefing, audit trail, and verbatim quotes were used to ensure that participants' personal perceptions were analyzed, and not the personal beliefs of the investigators [33]. Participants' quotes were translated by the first author and a research assistant with Bachelor's degrees in the English language.

Data were analyzed continuously until the point of saturation was reached. The sample was considered saturated when no new themes emerged from the gathered data [34]. The interview was adjusted twice, to increase insight into some of the new topics which arose from previously analyzed data. After interviewing 21 participants, data collection stopped, having reached saturation. Therefore, we concluded that the sample size of 21 was sufficient for an appropriate understanding of the topic and for answering the research questions "*how*" and "*why*".

### Measures for validity

The first author, who did the interviews, has worked in a rehabilitation clinic for 12 years, and so was familiar with the phenomenon of chronic pain management. This experience made communication with the participants easier. On the other hand, having been a therapist for many years, it seemed more difficult to not act as a therapist, which obviously wouldn't be the appropriate role within the context of interviewing. To avoid this risk, feedback on objectivity was given by an experienced psychologist during three pilot interviews, conducted independently from the study sample. In a further attempt to minimize the risk of observer bias, the interviews were also analyzed by the second author. The background of the second author, who had a great deal of experience in work and health research, was complementary to the knowledge of the first author. This gave us the opportunity to analyze the data from different perspectives.

## Results

**Figure 1:**
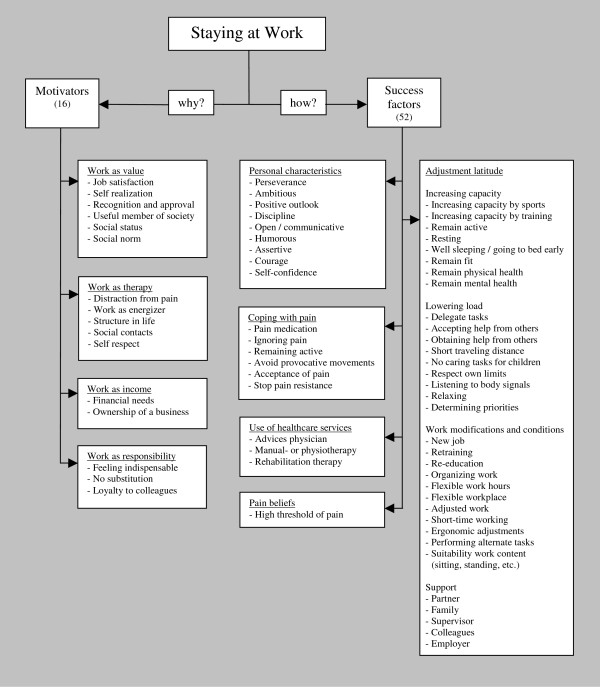
**Thematic content of motivators and success factors for staying at work with chronic pain**.

Several themes emerged after thematic analysis of the transcribed interviews. A total of four themes of motivators (work as value, work as therapy, work as income generator and work as responsibility) and five themes of success factors (personal characteristics, adjustment latitude, coping with pain, use of healthcare services, and pain beliefs) were recorded in the interviews. Within the group of participants the answers turned out to be divergent. Figure 1 outlines the categories of motivators and success factors for SAW that emerged after analyses of the interviews. In addition to motivators and success factors, two other themes in the interviews were: "Consequences of SAW" and "What can be learned from workers who 'stay at work' with pain."

### Motivators for staying at work

#### 1. Work as a value

In their work, participants found recognition and approval, self-realization, and self-respect. In a way, work gave meaning to the lives of many people. Participants stated that work provided a goal or mission in their lives.

*Job satisfaction *was often stated as a strong motivator for SAW. Work gave satisfaction because it was rewarding. Job satisfaction was linked to most other motivators listed in Figure 1. P3: "My work is wonderful; it gives me energy and satisfaction. I like my job and I don't want to lose it; it gives me the strength to continue working." On the other hand, some participants indicated that work was no longer giving them any joy, yet they kept on working. In those cases, other motivators compensated for this, for example, work as a means of ensuring income.

##### Self-realization

Some participants stated that they feared a stationary situation without their work. P18: "What would I do when the children are at school? I want to develop myself, learn new things, keep my mind active!"

##### Recognition and approval

At work participants felt valued and approved by others. P12: "In my job I get appreciation for what I do. That's why I work." Or another example: "My husband is always away on business, so it feels good to be with other people and to share common goals."

##### Useful member of society

Many participants felt an urgent need to participate in society. They feared losing touch with work and society. P6: "Everyone has to contribute to society, and I want to do my part. It's no use just being at home with my back pain, turning my back on society."

##### Social status

Having a job was regarded as status, making it evident that one can earn one's own living. P8: "Unemployment leads to social decline, which would be horrifying to me."

##### Social norm

Some participants try to act in accordance with what is the general belief. P8: "I think work is the norm." P15: "A man has to earn a living for his family; this is how things are meant to be." Fulfilling this ambition secured a feeling of self-respect.

#### 2. Work as therapy

Many participants experienced their work as being a place for healing and recovering. They indicated that work increased their mental and physical well-being.

##### Distraction from pain

Work distracted from the pain. P17: "Working gives me pleasure; often I am in a flow, forgetting the pain completely. At home there is a stimulus-deficit; there is no distraction, which intensifies my pain." And P2: "When I am busy and concentrating on my work, I have no time for pain. Other things become more important than pain." A few participants indicated that the harder they worked, the less pain they experienced. P4: "The harder I work, the less attention I pay to my pain." Some managed to go on acting this way at home too; others felt exhausted and had no choice but to lie down and rest.

##### Work as an energizer

Many participants felt work to be a source of new energy. P11: "When I work, I have more energy, even at home. I simply feel much better when I am working. One has to realize that work, whether with modifications or not, can act as powerful energizer."

##### A structure in life

A few participants found that work enabled them to live a life not constantly dominated by pain. P7: "It helps to organize your life and have control over the pain."

##### Social contacts

Being at work generated social contacts; it may prevent feelings of loneliness. P17: "My work helps me to escape from the daily routine at home. Most of my friends who live nearby are at their work and not available for socializing." Participants linked social contacts to distraction, indicating that contacts distracted them from the pain. P12: "I need some people around to talk with, to share common interests. At home I lack the opportunity to meet other people." Being around colleagues gave new energy.

##### Self-respect

Some participants mentioned that working brought about self-respect, a reason to be proud. P10: "Being at work again gave me a sense of belonging to society; it increased my self-respect." In this context, increased self-confidence has also been mentioned as a therapeutic aspect of working. P16: "I found out that I can do more than I thought I could, and that working doesn't make things worse."

#### 3. Work as income

##### Financial needs

For most participants a secure income appeared to be a strong motivator to stay at work with CMP. P13: "I feel the need to stay at work, because I am a breadwinner, and without my income we would have to sell our house." For others, the financial aspect was of less importance.

Being the *owner of a business *was mentioned as a strong motivator, for the obvious reason that work guaranteed income. P5: "If I didn't have a business of my own, there would be moments of short-time work disability." Employees qualify for workers' compensation/disability benefits. Owing to the high cost of insurance, self-employed people are seldom covered against illness. Moreover, self-employed participants were convinced that their commitment was keeping them at work.

#### 4. Work as responsibility

##### Feeling indispensable

A few participants felt they were indispensable at their work. They perceived their presence at work as a necessity, making them determined to work despite pain. Being absent without anyone *substituting *would mean that the work would not be done. The consequences of this could be very bothering: students would be deprived of education, patients wouldn't get the care they needed, deadlines would not be met, and productivity would drop. Most participants had strong feelings of responsibility and kept on working despite pain.

##### Loyalty to colleagues

Some participants felt that, by staying away from work themselves, their colleagues would have no other choice but to work harder to make up for them. Loyalty to colleagues appeared to be a significant motivator for SAW.

### Success factors for staying at work

Success factors for SAW were categorized into five theme groups: personal characteristics, adjustment latitude, coping with pain, use of healthcare services, and pain beliefs.

#### 1. Personal characteristics

Participants found their own characteristics to be an important success factor in SAW. Many times *perseverance *was mentioned as a success factor, indicating that SAW was not always easy. A few participants thought of themselves as *ambitious*, which was indicated as success factor for SAW. P1: "I really have the drive to be successful in my job; the pain is not going to stop me." A *positive outlook *was seen as an important factor for SAW. P6: "Take your chances, there's always something you can do. I'm inclined to look for opportunities instead of problems. If you can't climb the mountain, then travel around it to reach your goal." And P7: "Sometimes it's hard, but something negative should be turned into positive. Find ways to do what you want to do. Focus your mind on possibilities." Being *communicative, assertive*, or *self-confident *helped participants to ask for support, to set their limits, to balance load and capacity, to communicate their needs to the employer, and to initiate work modifications.

#### 2. Adjustment latitude

A large majority of the participants mentioned adjustment latitude as a powerful success factor for SAW. The possibility to balance working hours, workplace, and work pace gave participants the opportunity to organize their own work, and perform work tasks in accordance with their own conditions. P9: "I am in a fortunate position that I can determine my own workplace. Since I have a mobile phone, I am no longer forced to sit at my desk the whole day; I can move around now." Since not every workplace offers a high adjustment latitude, it seems likely that good working conditions contribute to a successful SAW. In order to create a balance between (work)load and capacity, participants had changed their behavior: they delegated tasks, accepted help from others, they complied with perceived physical and mental limits, organized work, and determined priorities of their own. On the one hand, they *increased their capacity *by participating in sports and training, remaining active as well as resting more frequently, or improving the quality of their sleep. On the other, their *load was lowered *by delegating tasks, accepting help from others, shortening the traveling distance to work, delegating child care, respecting their own limits by taking notice of their body signals, relaxing, and lastly, by determining their priorities. Participants organized *modifications at work *and suitable *work conditions *to decrease the work load. It appeared that modifications at work enabled workers with CMP to stay at work, and that these modifications were made at different levels: by changing jobs, by retraining to fulfill alternative tasks, and by organizing their work in terms of flexible working hours, more flexible work, adjusted work, short-time working, ergonomic adjustments, or having a more suitable work content. P2: "I have always worked in nursing, but now I am housekeeping for others, which is less demanding." And P9: "In our company there was a vacancy. This gave me the opportunity to find a more suitable job." P10: "If I had had to go back to slaughterhouse work, I would have been on sick leave again very soon." Or P17: "I worked in a nursing home, was always very busy. After work I used to be exhausted. At my new job things go better; when I'm home I have enough energy to do things for myself again." To some of the participants *suitable work content *was felt to be of upmost importance for SAW. A job requiring long stretches of standing in an upright position is not fit for a person who has difficulties with standing, while others indicate the opposite. P15: "If I'd have a job that would include a lot of sitting down, I'd have quit a long time ago."

*Support *from others was often mentioned as a success factor for SAW, for instance, with the spouse or children taking over housework, the extended family helping with the babysitting, and the manager or the employer allowing flexible working hours. P18: "My supervisor is very cooperative: as long as I work my hours and perform well, he doesn't care when or where the work is done." And colleagues relieved the workload. P15: "I've found a balance between what I can do and what not. In case of a strenuous project, I ask my colleagues for help; it has never been a problem for them."

#### 3. Coping with pain

Participants reported a variety of styles in coping with pain: some promoting and others hindering SAW. The effect of *pain medication *varied. Sometimes medication was considered to be a success factor for SAW. P21: "Without my medication I wouldn't be able to work; it's as simple as that." Pain reduction as a result of medication resulted in better sleep during the night. P4: "If I have a lot of pain, I take medication before sleeping. Next day I feel better." And it was experienced as facilitating a positive outlook. P17: "Don't think it is good or bad: just use them. Because when the pain is lower, you feel better, and more positive." On the other hand, some participants feared they might ignore pain signals and fail to take a break as a result, or they feared drowsiness, and the consequential disability in working. P1: "I don't want pain medication, because then I no longer feel my limits. I have decided I don't want to live a life on drugs. The pain is there, but it is bearable." These participants showed reluctance towards pain medication. Moreover, in many cases the pain medication did not help soothe the pain at all. Another reason for discontinuing pain medication was the notion of overcoming the pain on their own, taking responsibility, and no longer depending on someone or something else. *Ignoring the pain *was mentioned by some participants as a strategy to control the pain. Other participants *avoided provocative movements*, carefully taking notice of the pain and understanding the heightening pain level as a signal to stop overworking themselves. P9: "Listening to body signals and preventing overuse, and thus maintaining the balance, is what's keeping me going." *Remaining active *was experienced as promoting coping style for SAW by most of the participants, preventing deconditioning. P1: "I'm convinced that the best remedy for the pain is to remain active and to keep moving. It is keeping me fit." Many participants stressed that *pain acceptance *was a successful strategy to stay at work. P10: "You learn to accept the pain, to endure it, and to live with it." Putting an end to *resisting the pain *was considered to be in a direct line with pain acceptance. P3: "My girlfriend stays at home until the pain has gone; she can't accept doing things while having pain."

#### 4. Use of healthcare services

Although some participants indicated that they were disappointed in healthcare, others stated that healthcare services helped them to stay at work. Reassuring advice by physicians to keep exercising despite pain, manual therapy or physiotherapy, and rehabilitation therapy all made it easier for participants to stay at work. P12: "Twice a year I visit my physiotherapist for a couple of weeks. Without his help, I believe working would be practically impossible." Or P7: "During rehabilitation I learned to no longer let the pain become the main focus in my life. Doing the things I want to do, despite the pain, that helped me a lot. I even feel less pain."

#### 5. Pain beliefs

Most participants evaluated their *threshold of pain *as above average, which enabled them to act despite pain. P3: "People with higher thresholds of pain are able to tolerate their pain. They can do more with their pain, I guess." For a plausible explanation of how this higher threshold of pain came about, participants referred to the length of time they had had the pain. P17: "I've had the pain so long now, I'm used to it." A few participants said their threshold of pain diminished as the pain lasted longer. P10: "In the past I never needed painkillers at the dentist's; now I really can't do without. I have become more sensitive to pain."

#### Consequences of SAW

Participants stated that SAW had both positive and negative consequences. Most motivators for SAW were labeled as positive (Figure 1), and were seen as stimulants for participants to stay at work. But there were perceived negative consequences of staying at work despite pain. Diminished capacity for spare time activities such as sports, gardening, or social events was one. P17: "I go to bed early, to recover from my work and become fit again for tomorrow's work. There are hardly any opportunities for social activities." Or P14: "Gardening is fatal. Afterwards I'm dead beat; I can't even walk." A negative effect on one's private life and decreased quality of work was another. P20: "It is so much harder to concentrate when pain intensity is high." And then there was fatigue. P5: "The first thing I do after work is fall asleep on the sofa." And there was also frustration. P4: "Some colleagues of mine call in sick when they have a cold, which I find very annoying." And, finally, there was an increased level of pain.

#### What absentee workers can learn from the workers who stay at work with pain

In the interview a standard question was: "What could other workers with CMP, who are on sick leave, learn from you, so that they will be able to continue working?" Most participants were able to answer this question, revealing their personal success factor in the process: "Listen to your body language (what is your back trying to tell you?), take a rest when needed, stay active/keep exercising despite the pain, retrain for a more suitable job, get to work and don't give up, make something that seems negative into something positive, concentrate on possibilities instead of impossibilities, find a new job with less strain, set your own limits and be assertive, have the courage to change, keep yourself involved in society, go out of the pain and leave it behind, don't worry about the pain, learn to accept your pain, don't resist the pain, find a way to self-confidence, re-organize your life and seek help."

## Discussion

### Why

The first research question in our study, "Why do workers with CMP stay at work despite pain", resulted in four themes of motivation: work as value, therapy, income, and responsibility. Participants who stay at work with pain placed a high value on working. In general, participants in our study felt the need to stay at work, which seemed to encourage them to find ways to be able to stay at work. Feeling the desire to stay at work may be recognized as being an important success factor and a prerequisite for SAW despite pain. Strong motivation helped to strive for aims in life [27,29]. Participants in our study were willing to change in order to reach their goal: staying at work. A strong motivation to stay working set off behaviors such as increasing adjustment latitude, improving pain-coping strategies, organizing work modifications or better working conditions, accepting healthcare treatment, and seeking support. Contrary to what was investigated in our study, workers who were not able to stay at work may have other motives in life, overpowering the motivation to stay working [27,29]. For example, in a study on the question of deciding whether to work or to call in sick, participants chose calling in sick when they felt their daily lives had been affected in a bad way by their effort to stay working. Some chose to look after the family, instead of SAW [35]. Even so, there is a possibility that many absentees did have the intrinsic motivation to stay at work, but failed to find or put into practice the strategies needed [27,36]. People without the appropriate motivation or personal characteristics for promoting coping are bound to have more difficulties finding or developing strategies promoting RTW or SAW. Unfortunately, it is not at all easy to have a positive and optimistic attitude. It is obvious, however, that many success factors identified in our study can be put into practice by the workers themselves; yet sometimes people need help in finding alternative behaviors in order to stay working. We may be able to learn from the successful workers in our study, who have pointed out the essentials of staying at work. The results of this study could possibly be used to develop programs for sustainable RTW or as a guideline in attendance motivation. Strategies and competency leading to SAW can be trained or taught.

### How

The second research question, "How do workers with CMP stay at work", resulted in five themes of success factors: personal characteristics, adjustment latitude, coping with pain, use of healthcare services, and pain beliefs. Linton and Buer have suggested enlisting the help of workers who managed to successfully cope with CMP to "teach" their absentee colleagues. It appeared, however, that these successful workers only had a few suggestions and often were not fully aware of their own coping strategies [37]. In our study we asked: "What can others learn from workers who stay working with pain?" Most participants appeared to self-manage their challenges, take responsibility for themselves, and scored high on self-efficacy, although this did not always mean that they had been acting entirely on their own. Sometimes help had been offered and accepted from others. Participants had taken upon themselves the responsibility to change and had taken chances at the appropriate moment. Participants' personal characteristics had contributed to the power of self-management. However, self-management can also be taught. Acquiring these skills could be an ingredient of RTW programs to achieve sustained work participation. The successful strategies for SAW revealed in our study may be used for guidance.

This self-manager profile presented above resembles the profile of "Adaptive Copers" described by Turk and Rudy, characterized as experiencing low affective distress, high levels of daily activity, and locus of control [38]. The hypothesis that workers who stay at work act as "Adaptive Copers" needs to be tested using the Multidimensional Pain Inventory [39]. Boot and colleagues have distinguished four profiles of adaptation to functional limitations in workers with asthma and COPD: the eager, the adjusted, the cautious, and the worried [40]. These adaptation profiles provide insight into the different ways workers with CMP are coping with their pain at work. Adjusted workers have managed to adapt well to their limitations by finding the balance between workload and capacity. Eager workers are highly motivated to stay at work; they do not talk about the pain and perform well at work [40]. Participants in our study mostly resembled the Adjusted-worker profile and the Eager-worker profile. In a review of Shaw and colleagues, prominent work disability risk factors were identified, resulting in three high-risk profiles for prolonged work absence and disability: the immobilized, the disemployed, and the overwhelmed [41]. Success factors experienced by workers who stay at work in our study match the opposites of these different profile types for work absence and disability.

The themes of motivation "Work as income" and "Work as responsibility" have also been described in the Illness Flexibility Model by Johansson and Lundberg [42]. In this model, attendance requirements (negative consequences of being absent) experienced by workers were economic loss, accumulating work tasks, or unattended patients or students [26,36]. In addition, the latitude of workers for balancing work and capacity is recognized in the illness flexibility model, defined as adjustment latitude [42]. A high adjustment latitude "provides opportunities to work despite ill health." [36]. It has been concluded in the literature that a low level of adjustment latitude at work may mean a risk factor for sickness absence [43]. Other studies show that modifications at work for employees with work disabilities lower the levels of absenteeism [44,45]. Our study supported these findings: participants reported that moderation of work, making it more suitable to their capacity, turned out to be an important factor in SAW. Sometimes, there are obstacles within an organization that hinder a successful implementation of modifications in work [32]. Therefore, working conditions had better not be ignored and should be regarded as success factors for SAW [46]. The skills of successful participants in our study could be a helpful tool in programs preventing absenteeism. The extent of adjustment latitude at work should be taken into account as a possible risk factor.

In our study, it seemed that the lives of participants who "stay at work" despite pain were not dominated by the pain. The pain had not been "conquered," but accepted. This may explain why participants reported only a moderate level of work disability, while the pain intensity had been substantial (Table 1). In present-day pain management programs, pain acceptance is increasingly achieved by paying less attention to the pain by focusing on themes that are really important in life [47,48]. Clinicians could make use of the acceptance and commitment therapy (ACT) [47] in their treatment of workers with chronic pain, to help them to stay at work. Furthermore, the approach of acceptance may be valuable within an RTW program. Disabled workers may become conscious of work as being an important life value, which perhaps they hadn't realized before. This is supported in a recent study using the context of a work rehabilitation trajectory, by transforming the meaning of pain to facilitate RTW. Many workers were ready to accept the idea that the pain might never disappear and they were willing to learn how to deal with this reality [49].

Many explanatory models and theories have been created to understand and explain sick leave and work attendance. Sick leave has been linked to motivation [26,27,29,42,50,51], to stress and coping [25,52], to the balance between work demands and capacity [28,31], to adaptation [30], or to a combination of these [32]. All these models explain or predict the behavior of workers. At this moment it is unclear which model is the most appropriate one to explain SAW. It is difficult to fit all self-experienced determinants for SAW retrieved in our study into one of these models, although models which stress the multi-causality of work participation seem to be the most suitable. The model of the International Classification of Functioning, Disability and Health (ICF) could be used as a framework when categorizing the determinants, but it offers no explanation [53]. Our results indicate that a variety of factors are relevant for SAW, which is in accordance with the disability prevention management model of Loisel and colleagues [32]. In finding an explanation for sick leave or SAW, the compensation policy and social security system should be taken into account [54]. Recent studies indicate that in addition to workers' personal factors (workers' characteristics, health, and medical care), environmental factors such as job characteristics, work modifications, involved stakeholders, and the compensation system are of upmost importance for SAW and RTW [54-56].

### Strengths and limitations of the study

Qualitative research has certain pitfalls. To manage these, we have chosen measures offering valid and reliable results and conclusions. Attention was paid to credibility, transferability, dependability and confirmability [33,57]. To increase credibility and transferability of the results, we have created a varied sample. Unfortunately, in our study we had to do without participants between the ages of 20 and 30. Moreover, the majority of participants had experienced pain for more than five years. In the course of these years they may have learned to adapt to the pain and its limitations. As a result, the conclusions may be less suitable for generalization in cases of younger people and cases of people with a shorter history of pain. Since workers aged over 45 years more frequently call in sick, our sample still seems to be representative [3,58]. In the Netherlands the system of social security is a relatively generous one. Nevertheless, compared to working populations in other European countries, the amount of people working despite their pain is very similar: an average of 74% indicated that pain did not interfere with employment [5]. Furthermore, in the Dutch compensation system no distinction is made between work-related and non-work-related injuries. Therefore, in the analysis of this study no such distinction was made. Whether generalization of these results to countries with less generous social security systems is possible is uncertain: the relative weight of financial incentives may be stronger. The strength of our study is that it offers an overview of many motivators and success factors for SAW as experienced by participants with CMP. Because of the aim of our study, which was to learn from a successful group of workers, we selected workers who worked despite CMP. Apparently, their pain did not lead to strong disability; the score on the occupational parameter of the PDI was on average 3.7 (scale 0-10), indicating an almost moderate level of occupational disability. Therefore, the results of this study cannot simply be generalized to RTW populations. A comparison with workers who were not able to stay at work would have lent more weight to the results.

### Recommendations

We recommend using the experiences of our participants who revealed which factors and strategies were essential to them for staying at work with chronic pain. These success factors may offer some guidance in developing intervention programs to prevent absenteeism or to promote sustainable RTW. Research should not focus solely on characteristics of the individual workers but should also take into account contextual factors such as work environment, social security system, social situation, and healthcare system.

## Conclusions

Participants in our study experienced many motivators and success factors for SAW. Personal characteristics, well-developed self-management skills, and the drive to work may be seen as important success factors and prerequisites for SAW. Those behaviors promoting SAW were: increasing adjustment latitude, improving coping strategies, organizing work modifications, making use of healthcare services, and asking for support. These behaviors are modifiable and can be influenced by the workers themselves. Therefore, the results of this study may be used to develop preventive interventions to avoid absenteeism. Behavioral changes and competency resulting in SAW can be taught or trained by a clinician. Interventions to help workers to stay at work or return to work should take into account both the individual worker, as well as his social situation and work environment. Working conditions, such as flexible work hours or workplaces with high adjustment latitude, may be helpful for avoiding absenteeism. With a view to future research on work participation in CMP, it is recommended that the experience of those workers who have revealed which factors and strategies were essential for them to stay at working be used.

## Competing interests

The authors declare that they have no competing interests.

## Authors' contributions

HdV, SB, MR, JWG, and JG participated in the design and conception of the study. HdV conducted the interviews. HdV and SB read, coded, and analyzed the data. MR checked the coding and analysis. SB and MR helped to draft the manuscript. All authors read, revised, and approved the final manuscript.

## Pre-publication history

The pre-publication history for this paper can be accessed here:

http://www.biomedcentral.com/1471-2474/12/126/prepub

## Supplementary Material

Additional file 1 **Semi-structured interview**. These questions guided the interviews. Click here for file
