# Supplementary material for: Staying at work with chronic nonspecific musculoskeletal pain: a qualitative study of workers' experiences
Source: BMC Musculoskelet Disord. 2011 Jun 3;12:126. doi: 10.1186/1471-2474-12-126 (PMC3121659; doi:10.1186/1471-2474-12-126)
Supplement: Additional file 1 — Semi-structured interview. These questions guided the interviews. [file 1471-2474-12-126-S1.DOC]

**Additional file 1: Semi-structured interview**

General introduction

1. Which subjects, related to working with pain, would you like to talk about during the interview?

2. What kind of work do you do?

3. What kind of pain do you have?

Pain-related questions

4. What do you think is the origin of your pain?

5. How does the pain influence your life?

6. How do you cope with the pain?

7. How was pain-coping in your family when you were a child?

Work-related questions

8. What does work mean in your life?

9. What are reasons for you to work?

Work- and pain-related questions

10. Why are you working despite your pain condition?

11. What made you decide to continue working with pain?

12. Did you ever consider stopping working because of the pain?

13. What does it mean for you to be working with pain?

14. What consequences does working with pain have for you?

15. Are there moments you are tempted to call in sick?

16. What keeps you going at such moments?

17. Does the pain influence your productivity?

18. Does the pain influence your performance?

19. What has contributed to your staying at work?

20. Did you have to give up other aspects of life?

21. How do you manage working with pain? (What are your success factors for working with pain?)

22. What qualities do you have for continuing to work with pain?

23. What do you do to prevent absenteeism?

24. What was the best advice that helped you to stay working?

25. Why are you able to work with pain, while some other people are not?

26. How do you judge your future work situation within the next two years?

27. What can other workers with chronic pain, who become sick-listed, learn from you in order to stay working?

28. How did others contribute to your staying at work?

29. What was the role of healthcare services in staying at work?
